# Laboratory Demand Management Strategies—An Overview

**DOI:** 10.3390/diagnostics11071141

**Published:** 2021-06-23

**Authors:** Cornelia Mrazek, Elisabeth Haschke-Becher, Thomas K. Felder, Martin H. Keppel, Hannes Oberkofler, Janne Cadamuro

**Affiliations:** Department of Laboratory Medicine, Paracelsus Medical University Salzburg, A-5020 Salzburg, Austria; e.haschke-becher@salk.at (E.H.-B.); t.felder@salk.at (T.K.F.); m.keppel@salk.at (M.H.K.); h.oberkofler@salk.at (H.O.); j.cadamuro@salk.at (J.C.)

**Keywords:** pre-analytical phase, overutilization, underutilization, appropriate laboratory test ordering

## Abstract

Inappropriate laboratory test selection in the form of overutilization as well as underutilization frequently occurs despite available guidelines. There is broad approval among laboratory specialists as well as clinicians that demand management strategies are useful tools to avoid this issue. Most of these tools are based on automated algorithms or other types of machine learning. This review summarizes the available demand management strategies that may be adopted to local settings. We believe that artificial intelligence may help to further improve these available tools.

## 1. Introduction

Laboratory tests are fundamental for medical diagnosis, prognosis and treatment decisions [1] and are being ordered in rising numbers each year due to increased availability, mostly based on technological advances [2]. However, due to this fact that laboratory orders increase along with convenient availability, it seems that a certain amount of laboratory tests are ordered inappropriately [3,4]. On the one hand, inappropriate orders may present as overutilization, where tests with doubtful contribution to further patient management are ordered; on the other hand, there may be underutilization, when required tests are not being ordered [5]. Even if studies estimating over- or underuse are rarely comparable due to differences in study design, it seems that the extent is not negligible. In a systematic review, Zhi et al. [5] estimated an overall mean rate of overutilization of 20.6%. Subgroup analysis revealed a higher mean rate, around 44%, for inappropriate initial testing. However, single studies state that up to 70% of ordered tests may be of doubtful importance for patient management [6,7]. A workup of closed malpractice claims conducted by Gandhi as well as Kachalia et al. [8,9] revealed that failure to order the appropriate diagnostic or laboratory test contributed to missed or delayed diagnoses in 55% and 58% of cases in an ambulatory setting and the emergency department, respectively. Zhi et al. [5] state the overall mean rate of underutilization is 44.8%.

Along with Sarkar et al. [10], who support the high proportions of errors in test selection by evaluating orders for coagulation disorders in real time, inappropriate ordering may be considered as a substantial threat to patient safety. Overutilization may lead to unnecessary follow-up investigations or treatments, increased workload and costs as well as patient anxiety, while underutilization may result in missed or delayed diagnoses [5,11,12]. Lack of knowledge, insecurity, pure habit, patient pressure or fear of lawsuits are possible causes for inappropriate testing [13,14,15]. The lack of knowledge is reflected by various studies, which observed inappropriate orders despite available guidelines or recommendations on the implementation of demand management (DM) tools [12,14,16,17,18].

This review summarizes available DM strategies, which may be implemented into local settings to reduce inappropriate test utilization.

## 2. Possible Strategies to Avoid Inappropriate Test Utilization

**Figure 1:**
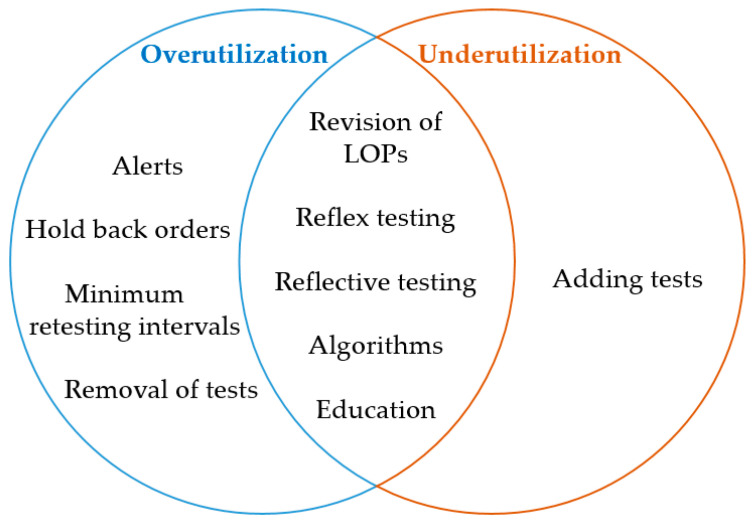
Categorization of DM strategies: Stratification of whether DM tools prevent overutilization and/or underutilization. LOP = laboratory ordering profile.

DM tools may help to prevent overutilization and/or underutilization. An attempt to categorize the different DM strategies as appropriate tools to overcome over- and/or underutilization is depicted in Figure 1.

Many studies combine several tools [14,17,19], which has been shown to have an additive effect on the overall outcome [20]. In addition, the collaboration of laboratory specialists and clinicians together with audits, feedbacks, reminders and multiple plan-do-study-act (PDSA) cycles will further improve efficiency in terms of a continuous improvement process [12,14,18,19].

### 2.1. Alerts at the Stage of Order Entry

Alerts appearing in the form of pop-up windows in the clinical physician order entry (CPOE) system may be designed to avoid various causes of overutilization.

Lippi et al. [21] implemented alerts for biological implausibility concerning age (e.g., beta human chorionic gonadotropin in patients <9 and >60 years) or gender (e.g., prostatic specific antigen (PSA) in females) at two university hospital wards. In addition, alerts for minimum retesting intervals (MRIs) were implemented (see Section 2.3). The alert provides an explanation as to why the order is deemed inappropriate and enables the ordering provider to choose order cancellation or acceptance.

Similarly, Juskewitch et al. [16] implemented an alert, triggered by the concomitant order of erythrocyte sedimentation rate (ESR) and C-reactive protein (CRP) in a community health system. Again, the user is informed about the inappropriate request and has the choice to cancel ESR or to proceed with the order. The implementation of this DM strategy resulted in a 42% relative rate reduction of ESR/CRP co-ordering.

Alerts may also help to suggest an alternative test, as Parkhurst et al. [22] showed. The authors reduced genetic testing of methylene tetrahydrofolate reductase (MTHFR) by informing the ordering physician about the latest recommendations of MTHFR testing, including the suggestion of homocysteine as alternate test. In this study, the choice of overruling or adopting the suggestion was left with the user. Overall, there was a significant decrease of average monthly MTHFR tests from 12.93 per million patients in the year before the intervention to 7.08 per million patients afterwards.

Larochelle et al. [17] aimed to improve ordering of cardiac biomarkers according to guidelines for the diagnosis of acute coronary syndrome (ACS). As part of a multimodal intervention, including education and several changes in the CPOE system (see Section 2.3 and Section 2.4), a pop-up alert was introduced, triggered by the order of creatinkinase (CK) and CK-MB isoform (CK-MB), informing the user about the recommended indications for these tests.

MRIs, which may also be implemented in form of alerts at the stage of order entry, are discussed in Section 2.3.

### 2.2. Hold Back Orders in the Laboratory Information System (LIS)

Informing the ordering provider through alerts at the stage of order entry would be the preferred solution; however, it may not always be possible to reject inappropriate orders in the CPOE system due to technical issues. In these cases, orders may be screened for appropriateness upon arrival in the LIS.

Cadamuro et al. [23] selected the analysis of anti-PF4/heparin antibodies (HIT-Ab) as the objective for a so-called gatekeeping strategy. This test is used in cases of suspected heparin-induced thrombocytopenia (HIT), type II. However, before ordering the HIT-Ab test, pretest probability may be assessed with the 4T-score [24]. The four questions of this scoring system were incorporated into the CPOE system, and the appropriate answers had to be selected from a drop-down menu as a mandatory part of the HIT-Ab ordering process. Subsequently, the score was calculated automatically within the LIS, and depending on the result, the LIS rejected or submitted the order for testing. In the case of rejection, the ordering physician was informed of the probability of a positive HIT-Ab test being <2% and the possibility to overrule the laboratory’s decision. This intervention resulted in a reduction of HIT-Ab testing of about 50%, without jeopardizing patient safety.

Mrazek et al. [4], who aimed to collect cases indicating a relationship between availability and number of ordered tests, described an example, provided by Maria Salinas, where the LIS held back orders, in which at least four tumor markers (TMs) were requested concomitantly. The laboratory specialist then decided upon the appropriateness of the order in synopsis with the patient’s medical record. Samples of inappropriate orders are stored until the order necessity is clarified with the general practitioner. Three years after implementation of this DM strategy, annual requests containing 4 or 5 TMs declined by 66%.

MRIs, which may be considered as a subset of holding back orders, are discussed in the following section.

### 2.3. Minimum Retesting Intervals

MRIs are defined as “the minimum time before a test should be repeated, based on the properties of the test and the clinical situation in which it is used” [25]. Recommendations for MRIs are freely available, for example, from the collaboration of the Royal College of Pathologists, the Association for Clinical Biochemistry and Laboratory Medicine and the Institute of Biomedical Science [25]. MRIs may be implemented in the LIS, dependent on available technical possibilities. Salinas et al. [26] implemented an MRI in the form of a comment on the laboratory report. In case a ferritin test was re-ordered within three days of the last order for inpatients and three months for outpatients, the LIS rejected the request and stated an explanation in the comment, including the previous ferritin value. The results showed that 3.9% and 12% of requested ferritin were not measured in in- and outpatients, respectively.

A similar approach was used by Mrazek et al. [27], who implemented a MRI of 60 days for hemoglobin A1c (HbA1c) at one site of a University Hospital (Landeskrankenhaus [LKH]). Inappropriate orders were automatically rejected by the LIS, and an automatically generated comment explained the inappropriateness, stated the date as well as the result of the last HbA1c test and advised calling the laboratory if the analysis was needed in a special situation. This resulted in a decline of HbA1c measurements by 15.8%. After the implementation of the MRI, only 1.1% of ordered HbA1c were measured within 60 days, compared to 15% before the intervention. At another site, the Landesklinik St. Veit (STV), the MRI was implemented by educational measures only (see Section 2.10).

One drawback of rejecting tests in the LIS is that unnecessary blood collections may be performed for cancelled tests. Therefore, it would be favorable if the requesting physician is at least alerted in the course of order entry. Waldron et al. [28] implemented an MRI of 48 h for CRP testing. The ordering provider was alerted, but as blocking the order was not possible at the stage of order entry, the LIS rejected the test and provided an accompanying comment on the report. Requests within the MRI were only possible through direct consultation of a consultant microbiologist. Over one year, CRP requests dropped by 7.0%, and CRP tests analyzed decreased by 12.3%. The results of Larochelle et al. [17], who implemented a duplicate order pop-up warning for troponin re-orders within 6 h as part of a multifaceted approach, are discussed in Section 2.4.

Different outcomes are reported with regard to the reactions to the alert. As already mentioned in Section 2.1, Lippi et al. [21] introduced pop-up alerts with the possibility to override the rule for biological implausibility as well as MRIs for 15 different tests at two University Hospital wards. In the observational period of six months, 22% of the orders generated an alert and 77% of these tests were cancelled. Lapić et al. [29] implemented an MRI for inpatients at a university hospital for 53 tests. The pop-up alert gave information about the inappropriateness, referred to the date as well as the status of the previous test request and included the possibility to override the warning. In the observational period of one year, 106,780 orders, which accounted for 14.8% of all requests, violated the defined MRIs. The percentage of ignored alerts depended on the tests, but for high volume tests, including complete blood count, CRP, alanine-aminotransferase (ALT), gamma-glutamyltransferase (GGT) and total bilirubin, which accounted for 65% of alerts, the alert was ignored in >85% of cases. Therefore, outcomes may depend on the clinical setting and may not be generalizable.

Moyer et al. [30] implemented MRIs for ionized calcium (iCa), magnesium (Mg) and N-terminal pro brain natriuretic peptide (NT-proBNP) for intensive care unit inpatients. The alert at the stage of order entry did not only depend on the MRI, but also on the previous results for iCa and Mg. The iCa alert was triggered if iCa was re-ordered within 24 h and the previous iCa result was within the reference range. The pop-up alert informed the user about the date and result of the previous order, provided information about clinical situations in which iCa might still be indicated and left the choice to cancel the request or to continue with the order. In the latter case, an indication for the re-order had to be provided. Comparison of 90-day periods before and after the implementation of this DM strategy revealed a decrease in test numbers of between 28% for NT-proBNP and 48% for iCa. In a six-month period after the implementation, 6110 alerts were triggered, with the majority for Mg (5160). Overall, alerts were dismissed in 66% of the cases, again, with the majority for Mg testing (88%). iCa and NT-proBNP were re-ordered only in 5% and 7% of cases, respectively. Regarding patient safety, the authors examined the International Classification of Diseases Ninth Revision (ICD-9) codes, which may be associated with electrolyte disturbances. Despite the decline in electrolyte measurements, no increase of ICD-9 codes was observed.

Riley et al. [31] aimed to avoid duplicate genetic testing, as this is generally indicated only once in a patient’s lifetime. If the order has already been performed, the ordering provider was informed about the date of the previous result. Repeated analyses could be ordered by phone only. Evaluation after the intervention revealed that 82% of repeated orders were justified because the previous order yielded no result due to errors in the testing process. The authors mention that they have adjusted the programming according to these results, but this was not included in the study.

### 2.4. Revision of Laboratory Ordering Forms and Profiles

The position where tests are placed in the order entry system may affect the number of placed orders [3]. Furthermore, laboratory ordering profiles (LOPs), which are used to order a bundle of defined analytes with one click in the CPOE system, seem to be a source of overutilization; studies show that the number of orders drops after removing tests from such LOPs. An example provided by Michael Cornes describes a reduction of GGT orders of 82% after the test aiming to assess liver function was removed from the LOP [4]. Keppel et al. [32] retrospectively evaluated a DM strategy implemented to reduce unnecessary testing of the cardiac markers high-sensitive troponin T (hsTropT) and NT-proBNP. This intervention was conducted in collaboration with clinicians at three wards of the department of Cardiology, Clinic of Internal Medicine II, University Hospital Salzburg. The implementation started in one ward with an educational approach (see Section 2.10). Later, both cardiac markers were removed from all LOPs of the three wards, along with the distribution of information about the correct use of hsTropT and NT-proBNP in the form of guidelines and oral presentations. Despite the opportunity to order both tests without restrictions in the CPOE system separate from the LOP, monthly orders decreased by 66.1% and 75.8% for hsTropT and NT-proBNP, respectively, on all three wards. These results indicate that LOPs may indeed be a source of overutilization since they are often not used correctly (e.g., for specific indications) but merely for convenience purposes. Regarding patient safety, length of patient stay and 30-day all-cause re-admission rate were evaluated as surrogate markers, without adverse outcomes.

Similarly, Larochelle et al. [17] removed cardiac markers from LOPs. While CK and CK-MB were entirely removed, troponin remained in two LOPs for evaluation of new symptoms suggesting ACS. As indicated above, this DM strategy was implemented in a multifaceted approach. Altogether, the percentage of patients per month with guideline-concordant ordering of cardiac markers for ACS increased from 57.1% to 95.5%. Annually, ordered tests decreased by 16%, 87% and 95% for troponin, CK and CK-MB, respectively.

Along with educational sessions, audits and feedback, Bartlett et al. [14] introduced a panel for CRP and ESR testing. CRP was preselected, and an explanation referred to the recommended indications for these tests. Overall, ESR as well as combined ESR/CRP testing were reduced by 33% and 25%, respectively, while the mean number of CRP tests remained unchanged. However, further examination of patients’ charts revealed that inappropriate ESR orders remained after the intervention.

Other studies focus on LOPs for specific indications or diagnoses. Delvaux et al. [33] conducted a randomized controlled trial among general physicians (GPs). LOPs were created for 17 selected indications, based on available guidelines. In the intervention group, GPs received suggested analyses through the CPOE system after selecting an indication, and modifications were allowed before submitting the request. The control group also stated the indication of their orders but did not receive suggestions for test ordering. In the intervention group, the proportion of appropriate tests significantly increased by 0.21 for all tests. In the intervention arm, only 24 tests were ordered per panel, compared to 31 tests in the control arm. The evaluation of potentially delayed diagnoses revealed no difference between the groups. This is an example of how laboratory specialists may aid in test requesting and of how physicians are willing to accept their expert opinion.

Whiting et al. [12] aimed to standardize blood tests and introduced the possibility for primary care physicians to order “test groups” for monitoring patients with chronic diseases. Compared to previous habits, full blood counts (FBCs) and liver function tests (LFTs) were not required in this indication. The implementation comprised several PDSA cycles, educational sessions and regular meetings for discussion and feedback. Requests per 1000 patients significantly decreased by 14% and 22% for hemoglobin assessing FBC and bilirubin assessing LFT, respectively. Sodium, which was not affected by the DM strategy, and ALT values ≥ 120IU/L, which were assessed to identify alterations in possible significant pathology, did not show significant changes. Therefore, the authors concluded that the measures may not lead to more missed diagnoses.

In conclusion, LOPs should be revised to suggest appropriate tests for specific indications or diagnoses [34] rather than for unspecific “routine” panels.

### 2.5. Removal of Outdated Tests

Apart from giving an alert for inappropriate orders, tests may also be entirely removed from the order entry system. One example within the publication of Mrazek et al. [4], provided by Ana-Maria Simundic, refers to a stepwise elimination process of CK-MB isoform. According to an expert consensus document, CK-MB isoform may not be necessary in the case of high sensitive troponin assay availability [35].

### 2.6. Display Costs

Some studies evaluated the effect of displaying costs during the order entry process. Horn et al. [36] selected 27 laboratory tests, which yielded overall high costs due to the high price of a single analysis or to frequent ordering. Through the intervention period, the costs were displayed to primary care physicians of a group practice (“intervention physicians”), while physicians of other group practices, who received no information about prices, served as a control. In addition, the intervention physicians were informed about the aim of the project via e-mail. The results showed that for five of the twenty-seven tests, the display of cost information was associated with a statistically significant reduction in monthly laboratory ordering rates.

Similarly, Feldman et al. [37] focused on laboratory tests that were either frequently ordered or expensive. Different from the above-mentioned study, this intervention was conducted in a tertiary care hospital, and 61 laboratory tests were randomized, with the costs displayed (“active” arm) or not (control arm). The ordering physicians were not actively informed about why fees were displayed. This intervention resulted in a 9.1% reduction of orders in the active arm, while the orders of control tests increased by 5.1%.

Silvestri et al. [38] conducted a similar study in an academic health system comprising three hospitals. The evaluation of laboratory orders before and after the implementation of cost display for 1032 laboratory tests revealed decreased likelihoods for patients with orders during the encounter. Even if tests were ordered, the proportion of requests on a given hospital day as well as the number of tests ordered in one day decreased. In addition, in-hospital mortality, which was assessed for patient safety, did not increase in the post-intervention period.

Overall, the interventional impact was rated as “modest” by the authors [36,37,38]. Furthermore, investigation of appropriateness of test selection was not part of the study designs. Costs should never be the sole decision criterion for laboratory test ordering, not only because patient well-being should always be the number one priority of each physician, but also because laboratory costs only contribute to up to 2.5% of the overall healthcare costs but make up the majority of medical decision-making [39]. Therefore, cost reduction in the laboratory would have a minor impact on the total budget but a major impact on the quality of patient care. This opinion is supported by the survey of Horn et al. [36]. Only a minority of surveyed clinicians stated that cost information frequently influenced their decisions. In general, cost control is endorsed by clinicians, but reductions in expenditure may be also achieved by implementing DM strategies, which combat overutilization [14,16,17,19,21,26,28,29,30,31].

### 2.7. Adding Tests

Adding tests may be one attempt to prevent delayed or missed diagnoses. Salinas et al. [40] added calcium testing to orders from primary care patients older than 45 years of age and without a previous calcium test within the last three years. Using this approach, several cases of primary hyperparathyroidism could be detected.

### 2.8. Reflex and Reflective Testing

Another possibility of adding tests is through reflex or reflective testing. While reflex testing refers to the automated addition of tests according to a fixed algorithm within the LIS, reflective testing is the approach of adding tests and/or comments after the laboratory specialist has interpreted the results in synopsis with available clinical information [41].

In general, reflex testing may be used to prevent over- as well as underutilization. For example, reflex testing may be suitable for the stepwise analysis of thyroid hormones, where thyroid-stimulating hormone (TSH) is the initial test, and subsequent analysis of free thyroid hormones should only be performed in the case of abnormal TSH results [18,19,42]. By implementing this reflex, subsequent tests cannot be missed (preventing underutilization), and clinicians do not have to order all tests at once, which would be a source of overutilization. With regard to patient safety, retrospective data analysis may help to identify appropriate cut-off points for reflex testing [42]. Furthermore, two studies address the important topic of quality improvement and balancing measures. In the study of Taher et al. [18], continuous data monitoring and collaboration with clinicians resulted in improvements of the TSH reflex algorithm through two PDSA cycles. Gilmour et al. [19] accompanied the two-stage process by a root cause analysis, feedback through a survey and baseline data analysis to evaluate potentially missed diagnoses after the implementation of the reflex system.

Reflex as well as reflective approaches may also be combined as depicted by Elnenaei et al. [43], who describe an approach to early detection of pituitary dysfunction. Reflex rules for selected hormones were defined in collaboration with laboratory and endocrine physicians and implemented in the LIS. Lists of identified test results were then further evaluated by a laboratory physician, who decided if defined follow-up tests were indicated or not (reflective testing) based on previous results and available clinical information. In the case where added tests yielded abnormal results, the laboratory physicians would add an appropriate comment, including possible causes as well as a referral to an endocrine physician, if required.

Oosterhuis et al. [44] conducted a randomized controlled trial on reflective testing. The laboratory specialist performed reflective testing according to a routine procedure. Afterwards, it was randomly assigned whether the primary care physician received the added results or comments (intervention arm) or not (control arm). The evaluation of medical records revealed a significant positive outcome for patient management in the intervention arm.

In general, the evaluation of a questionnaire with clinical scenarios revealed that reflective testing is appreciated by physicians, but it depends on the tests and associated ethical questions. For pregnancy tests or PSA, physicians wish to be consulted before the tests are added. Furthermore, after adding tests it must be ensured that the results are not overlooked [45]. In a survey among patients attending a general practice surgery or hospital outpatient clinics, the majority endorse the concept of reflective testing [46].

### 2.9. Algorithms

Algorithms are an advancement of reflex und reflective testing; several concatenated if/then queries are addressed, until a diagnostic decision is possible [47]. One practical example of such an algorithm is the PTT Advisor, a mobile application that helps to choose the appropriate follow-up tests in patients with a prolonged partial thromboplastin time (PTT) and normal prothrombin time [48]. However, an evaluation of apps with regard to the impact on test ordering would be meaningful. Meyer et al. [49] propose an approach with patient vignettes that have to be solved either with the PTT Advisor or with usual clinical decision support. The results indicate a superiority of the PTT Advisor regarding test ordering and diagnostic decision-making. In addition, a questionnaire may be used to identify further fields of improvement of the mobile application.

Another way to implement diagnostic algorithms would be to program the according if/then cycles directly into the LIS. Furundarena et al. [50] introduced a new possibility for physicians to order an “initial study of anemia”. The order starts with the analysis of a hemogram, and only upon detection of anemia are further tests added automatically in the LIS according to predefined rules. Since the ordering provider does not know beforehand if the patient is anemic, follow-up tests are often ordered simultaneously with the hemogram. Therefore, many of these overused analyses were prevented with the algorithm, since anemia was only present in 20% of these requests.

Definition of these algorithms requires a lot of knowledge and time, as they need to be based on current evidence, with the need of annual revision. Additionally, evidence may not be available for every step or may be contradictory. In these cases, expert opinions would be the method of choice. Information technology (IT) aids in constructing these algorithms, and constant improvement in form of artificial intelligence solutions would be the next logical step.

### 2.10. Education

The impact of educational interventions in the form of a workshop for general practice trainees was assessed by Morgan et al. [15]. In two of three clinical scenarios, inappropriate testing was reduced after the workshop. However, in one scenario appropriate testing even decreased. A questionnaire, which was distributed, revealed that the attitudes of the trainees changed. The proportion of GP trainees believing that over-testing is a problem and tests can harm patients increased after the workshop, while fewer stated that they were reassured, felt pressured by patients and believed that they were less likely to be sued if more tests were ordered.

Other authors have used educational measures as the first step of a two-stage process. Gilmour et al. [19] aimed to reduce inappropriate free thyroxine (fT4) and free triiodothyronine (fT3) testing. In a first step, physicians were informed about the appropriate utilization of fT3/fT4 via oral presentations, emails and postings. In a second step, an automated reflex system was implemented, adding fT4 in the case of an abnormal TSH. FT3 was only available by providing a clinical justification. The median numbers of weekly free thyroid hormones did not decrease significantly from baseline (90 and 39 for fT4 and fT3, respectively) compared to the period after the educational intervention (78 and 34 for fT4 and fT3, respectively). However, after the implementation of the reflex system, fT4 and fT3 testing could be reduced significantly to a weekly median of 59 and 14 for fT4 and fT3 tests, respectively. In the study of Keppel et al. [32], which was mentioned in Section 2.4, the first step in reducing cardiac markers was to inform the medical staff of one of the wards about the recommended indications for NT-proBNP and ask the ordering physicians to deselect NT-proBNP from LOPs where the order was not indicated, resulting in a reduction of 52.8% of respective orders. After further removal of NT-proBNP from LOPs, the overall decrease was 84.6% of NT-proBNP tests per month at this ward. These studies used a stepwise approach to show that education may reduce the test volume, but the effect may be more pronounced by an additional implementation of IT-based solutions.

The observation that education as a sole method seems to be inferior compared to automated solutions was also noticed by our study group, comparing educational measures to an automated re-testing interval at two sites of a university hospital [27]. The implementation of the automated MRI for HbA1c in the LIS was mentioned in Section 2.3. In STV, only educational measures were realized, comprising the oral presentation of the evidence-based use of HbA1c in daily meetings and putting up posters to remind the medical staff about the re-testing interval of 60 days. Compared to the baseline period before the intervention, HbA1c measurements dropped by 21.1% after the educational measures in STV. The decline from 7.4% to 3.6% of HbA1c measured within 60 days was significant, but less pronounced compared to the automated MRI implemented in the LKH.

On the one hand, as already mentioned above, educational measures, e.g., transfer of information orally in several meetings or in a written form via pocket-sized cards or brochures, may accompany IT-based solutions to fill the gap of knowledge concerning appropriate utilization [12,14,17]. The effectiveness of education was not evaluated separately in these studies. On the other hand, IT-based solutions may also serve as a learning tool, as stated for the algorithm in form of a mobile application [49]. Lippi et al. [21], who implemented alerts for biological implausibility and MRIs, observed that the number of requests violating the rules decreased. Likewise, Waldron et al. [28] attribute the decline of CRP requests after the implementation of the MRI to an altered behavior of ordering providers. However, we could not observe an educational side effect of the automated MRI of HbA1c, implemented in form of a comment on the laboratory report. As already mentioned in Section 2.3, measurements of HbA1c significantly decreased after the implementation of the automated MRI, but the number of orders remained nearly unchanged in the LKH [27].

## 3. Discussion and Conclusions

There is broad approval that laboratory DM approaches are useful for appropriate test utilization, and several tools are already in use [51]. However, there are still a number of challenges. Due to different outcome criteria and settings, results may not be generalizable or comparable, which is why DM approaches have to be adapted to local settings. Therefore, harmonization strategies would be desirable. However, a survey conducted by the European Federation of Clinical Chemistry and Laboratory Medicine (EFLM) Working Group on Harmonization of the total testing process (WG-H) among national society members of the EFLM revealed that existing harmonization activities are not coordinated. MRIs are one example mentioned, for which the EFLM WG-H wants to start initiatives to produce official documents in European countries [52]. In addition, the third EFLM Strategic Conference under the chair of EFLM president Ana-Maria Simundic was planned to focus on DM only and to generate several task-and-finish groups that would lead the profession in this direction. Sadly, this conference had to be postponed due to the COVID-19 pandemic. We believe that the topics of harmonization as well as DM are recognized by laboratory specialists and that progress will be made over the coming decade.

As mentioned above, another challenge is that inappropriate orders remain [14]. One possibility for achieving appropriate test selection may be to conduct a health technology assessment prior to test implementation. Landaas et al. [53] describe an approach whereby the Laboratory Formulary Committee, comprising different medicine professionals, and the Smart Innovation staff of the local hospital analyzed a new molecular bladder cancer test according to a locally implemented health technology assessment program. In conclusion, the committee currently does not support system-wide use, but decided to start a small pilot study. The results thereof indicate that the test could have benefits for selected patients.

However, these evidence-based assessments and further recommendations proposed for successful implementation, like the selection of quality indicators for monitoring and improvement as well as the ensuring of regular updates, are time-consuming [54]. We believe that artificial intelligence (AI) solutions are the next logical step, aiding in the development as well as improvement of DM strategies, as they could help to manage large data sets. The synopsis of results from laboratory medicine, diagnostic imaging and pathology is necessary for the purpose of integrated diagnostics. Furthermore, the patient’s history, comorbidities, symptoms and treatments have to be taken into account for correct interpretation [55]. Currently, few published articles deal with the issue of applying AI algorithms to laboratory test selection. Islam et al. [56,57] have published two such studies, one of which in this issue, where they developed a deep learning algorithm based on retrospective patient data to predict appropriate laboratory tests. Xu et al. [58] aimed to identify superfluous tests in existing lab orders by estimating normal test results within a retrospective dataset. Machine learning (ML) models may also be used to identify prognostic factors. Tseng et al. [59] incorporated clinical, pathological and cancer-related gene features of patients with advanced oral cancer and found that only 6 of 44 genes analyzed are necessary for further prognostic risk stratification. Therefore, costs and resources for molecular analysis could be reduced with targeted requests. The MRIs mentioned above are implemented as pre-defined alerts, and various alert ignorance rates are discussed in Section 2.3. Concerning this challenge, Baron et al. [60] mention an approach where logistic regression models may be used to predict whether alerts will be accepted or overruled. The aim is to reduce the alert burden for the ordering clinician by showing only alerts that have a high probability of being accepted. However, not all questions can be solved with AI. For example, using serum tumor markers alone for cancer screening may currently not be recommended even if data were retrospectively evaluated using various ML models [61].

Furthermore, it has to be acknowledged that AI is only a tool of assistance [57]. A combination of computerized and physician-guided processes may be better than each one on their own. Wang et al. [62] proved this theory when evaluating the efficiency of a deep learning system and experienced pathologists in detecting breast cancer cells. AUROC values were 0.925 for the former and 0.966 for the latter, but 0.995 when combined. Therefore, AI solutions may complement the recommended collaborations with clinicians for successful implementation [54]. Intensifying collaborations should be a feasible task, since a survey indicates that interest from both professions exists [51]. An advantage of complementary AI solutions would be that these systems, fed with unfiltered patient data, are capable of finding completely new diagnostic strategies that humans have not yet thought of. Lien et al. [63] compared different ML models concerning the prediction of the two-day mortality of thrombocytopenic patients on the basis of hematological tests only.

In conclusion, the implementation of DM tools of laboratory specialists in collaboration with clinicians is increasing, and the incorporation of AI solutions is emerging in recent years. We believe that these solutions will help us to overcome technical barriers, a lack of harmonization and other challenges.

## Data Availability

No new data were created or analyzed in this study. Data sharing is not applicable to this article.
